# Molecular Epidemiology of Escherichia coli Clinical Isolates From a Tertiary Care Hospital in Uttarakhand, India: Insights Into Antimicrobial Resistance (AMR) Burden, Virulence Profiles, and High-Risk Lineages

**DOI:** 10.7759/cureus.102613

**Published:** 2026-01-30

**Authors:** Ranjana Rohilla, Mohit Bhatia, Varun Shamanna, Geetha Nagaraj, Pratima Gupta, Balram Ji Omar, Harshitha Gk, K L Ravikumar

**Affiliations:** 1 Microbiology, All India Institute of Medical Sciences, Rishikesh, IND; 2 Microbiology, Vardhman Mahavir Medical College and Safdarjung Hospital, Delhi, IND; 3 Central Research Laboratory, Kempegowda Institute of Medical Sciences, Bengaluru, IND; 4 Biotechnology, Nitte Mahalinga Adyantaya Memorial (NMAM) Institute of Technology, Nitte Deemed to be University, Karkala, IND

**Keywords:** antimicrobial resistance, escherichia coli, multidrug-resistant clones, multilocus sequence typing, virulence genes, whole-genome sequencing

## Abstract

Introduction

*Escherichia coli* (*E. coli*) is a major cause of extraintestinal infections and a leading contributor to antimicrobial resistance (AMR), particularly in low- and middle-income countries. The increasing convergence of multidrug resistance and virulence within high-risk clones poses serious challenges to treatment and infection control. Genomic data on circulating clinical *E. coli* lineages from India remain limited. This study aimed to characterize the population structure, AMR determinants, virulence profiles, and plasmid content of clinical *E. coli* isolates using whole-genome sequencing (WGS).

Methods

An exploratory study was conducted on 50 clinical *E. coli* isolates collected between January 2018 and October 2020 from diverse clinical specimens at a tertiary care teaching hospital in Uttarakhand, India. Antimicrobial susceptibility testing was performed using the VITEK 2 Compact system and interpreted according to the Clinical and Laboratory Standards Institute (CLSI) guidelines. WGS was carried out on the Illumina MiSeq platform. In silico analyses included phylogrouping, multilocus sequence typing (MLST), serotyping, AMR gene detection, virulence factor profiling, and plasmid replicon typing.

Results

Multidrug resistance was observed in the majority (45/50 (90%)) of the isolates. Universal phenotypic resistance was noted to fluoroquinolones (50/50 (100%)) and third-generation cephalosporins (50/50 (100%)), while carbapenem resistance was detected in 35/50 (70%). Tigecycline and colistin retained in vitro activity in 47/50 (94%) and 50/50 (100%) isolates, respectively. Genomic analysis identified extended-spectrum beta-lactamases (ESBL) genes in all isolates and carbapenemase genes (*bla_NDM-1_*, *bla_NDM-5_*, *bla_OXA-181_*) in 38/50 (76%), with high genotype-phenotype concordance. The predominant sequence type was ST167 (15/50 (30%)), followed by ST410, ST648, ST405, and ST131. Virulence profiling demonstrated predominance of ExPEC-associated adhesins (*fim*, *pap*, *afa*) and iron acquisition systems (*iutA*, *fyuA*, *iroN*). No classical diarrheagenic *E. coli* pathotypes were identified. IncF-type plasmids were the most prevalent.

Conclusion

This study highlights the alarming convergence of virulence and multidrug resistance among dominant *E. coli* clones in a tertiary care setting in India. The findings underscore the value of WGS-based surveillance in guiding antimicrobial stewardship and strengthening infection control strategies.

## Introduction

*Escherichia coli* (*E. coli*) is a ubiquitous Gram-negative bacterium that resides predominantly in the gastrointestinal tract of humans and animals. While most strains are harmless commensals, several have evolved pathogenic traits leading to a diverse range of infections, both intestinal and extraintestinal in nature. It is a leading cause of urinary tract infections (UTIs), respiratory infections, bloodstream infections (BSIs), meningitis, and wound infections globally, making it a critical pathogen in both community and healthcare settings [1,2].

The clinical burden of* E. coli* is particularly pronounced in developing countries, where limited diagnostic resources, irrational antibiotic use, and poor infection control practices contribute to the high morbidity and mortality associated with this organism. Furthermore, the bacterium is a major driver of antimicrobial resistance (AMR) in Gram-negative infections, including resistance to broad-spectrum beta-lactams, fluoroquinolones, and aminoglycosides. Increasing rates of extended-spectrum beta-lactamase (ESBL)-producing *E. coli*, and more recently, carbapenem-resistant strains, have been documented in both community-acquired and nosocomial infections, posing significant challenges to clinical management [2]. The Antimicrobial Resistance Surveillance Network (AMRSN), Indian Council of Medical Research (ICMR) has documented a total of approximately 99,000 culture-positive isolates from all clinical samples during the year 2023 from tertiary care hospitals across the country. *E. coli* was the most frequently detected bacterium across blood, urine, cerebrospinal fluid, and respiratory specimens. Analysis of antimicrobial susceptibility results revealed a progressive decline in *E. coli* sensitivity to several agents when compared to the year 2017. Susceptibility to piperacillin-tazobactam showed an approximate one-third reduction over the study period, while activity of amikacin declined modestly. Carbapenem susceptibility demonstrated a pronounced downward trend, with imipenem activity declining by nearly one-quarter and meropenem showing a smaller but consistent reduction. Fluoroquinolone activity was particularly compromised, with levofloxacin exhibiting the lowest overall susceptibility among all agents tested [3].

Infections caused by extraintestinal pathogenic *E. coli*, including uropathogenic *E. coli* (UPEC), neonatal meningitis *E. coli*, and sepsis-associated strains, are characterized by the presence of virulence factors such as adhesins, toxins, siderophores, invasins, and protectins that facilitate colonization, immune evasion, and systemic spread [4]. Genomic islands, including pathogenicity islands (PAIs) and high pathogenicity islands (HPIs), encode many of these traits and are often co-located with resistance genes on plasmids, thus compounding the threat [5]. The emergence of new pathotypes and plasmid-mediated gene transfer has further blurred the boundaries between classical E. coli pathotypes, resulting in increased clinical complexity [6].

Concurrently, the rising prevalence of plasmid-mediated resistance genes such as *bla_CTX-M_*, *bla_NDM_*, *bla_OXA-48_*, *mcr*, and *qnr* is of particular concern. These resistance determinants often reside on mobile genetic elements that facilitate their rapid dissemination across *E. coli* populations and to other *Enterobacterales *[7]. The combination of virulence and resistance has led to the global emergence of high-risk *E. coli* clones capable of causing hard-to-treat infections with significant healthcare implications. In India, studies have consistently reported high rates of ESBL and carbapenemase-producing *E. coli*, particularly from intensive care units and tertiary care hospitals [8,9]. Recent genomic studies have highlighted the emergence and dominance of specific multidrug-resistant (MDR) *E. coli* lineages that combine resistance and virulence. Whole-genome single-nucleotide polymorphism (SNP) analyses of *E. coli *strains revealed that ST167 has become the predominant clone, frequently carrying *bla_NDM-5_* and *bla_CTX-M-15_*, followed by ST405 and the globally disseminated ST131, while ST410 has also been identified as a rising lineage of concern. [10]

Despite the clinical significance of *E. coli*, relatively few studies from India have comprehensively examined the genomic profiles of clinical isolates using whole-genome sequencing (WGS). Traditional phenotypic assays provide limited insight into the molecular mechanisms underlying pathogenicity and resistance. Genomic tools such as WGS offer unprecedented resolution for pathogen surveillance, outbreak detection, resistance tracking, and characterization of virulence traits [11]. Platforms such as Pathogenwatch, Virulence Factor Database (VFDB), now enable rapid and comprehensive annotation of sequenced genomes [12,13].

Understanding the local epidemiology and genomic characteristics of *E. coli* clinical isolates is essential for guiding antimicrobial stewardship (AMSP) and infection control strategies. This is particularly critical in the Indian context, where antibiotic misuse, lack of surveillance infrastructure, and the burden of infectious diseases contribute to the evolution and spread of resistant pathogens. Genomic surveillance offers an integrated approach to mapping pathogen diversity, resistance mechanisms, and virulence potential and has been instrumental in informing global health policies [13,14].

This study used WGS to characterize 50 clinical *E. coli* isolates from patients at a tertiary care teaching institute in Uttarakhand, India. The objectives were to determine the phylogenetic background, sequence types, serotypes, AMR genes, plasmid profiles, and virulence gene profiles of the isolates. Through this genomic approach, we sought to provide comprehensive insights into the population structure and pathogenic potential of *E. coli* isolates circulating in tertiary care settings in India, thereby contributing to the growing body of genomic epidemiology data essential for informed public health action.

## Materials and methods

An exploratory study was conducted from January 2018 to October 2020 after obtaining ethical approvals from the Institutional Ethics Committees of the All India Institute of Medical Sciences, Rishikesh (IEC/18/477), and the Kempegowda Institute of Medical Sciences, Bengaluru (IEC/S12-2017). A nonprobability, convenience (consecutive) sampling strategy was employed. A total of 50 clinically significant *E. coli* isolates recovered during the study period were consecutively included without stratification by patient demographics, timing of collection, or AMR profile. The isolates were obtained from patients presenting with wound infections, urinary tract infections, lower respiratory tract infections, and pleural or peritoneal infections. This approach is appropriate for laboratory-based studies focused on phenotypic and molecular characterization rather than prevalence estimation. All confirmed isolates were subsequently forwarded to the Central Research Laboratory (CRL), Kempegowda Institute of Medical Sciences, Bengaluru, for further characterization and WGS.

At CRL, the isolates were revived and underwent preliminary identification and antimicrobial susceptibility testing (AST) using the VITEK 2 Compact system with the GN AST card (bioMérieux, Marcy-l’Étoile, France). Results were interpreted according to the Clinical and Laboratory Standards Institute (CLSI) 2024 guidelines [15]. Multidrug resistance (MDR) was defined as nonsusceptibility to at least one agent in three or more antimicrobial classes, and AST quality control was performed using *E. coli* ATCC 25922 in accordance with CLSI recommendations. DNA was extracted from the 50 *E. coli* test isolates using the Qiagen DNA Minikit (Hilden, Germany), and library preparation was conducted with the New England Biolabs ultraFSII (Ipswich, MA, USA). Quality control of the libraries was performed with Agilent TapeStation, after which sequencing was carried out on the Illumina MiSeq (San Diego, California, USA) using the v2 kit to generate 250 bp paired-end data. 

Assembly and quality control of the sequences were conducted using the GHRU assembly pipeline as detailed in protocols.io [16], developed by the National Institute for Health Research Global Health Research Unit on Genomic Surveillance of AMR, resulting in 50 whole-genome sequences. These sequences have been deposited in the European Nucleotide Archive under the study accession PRJEB29740.

ECTyper tool (v1.0.0) was used for in silico serotyping covering O and H antigens. Additionally, virulence factor gene analysis was performed using the Virulence Factor Database (VFDB) [17-19].

## Results

The 50 test isolates were obtained from various clinical samples, like pus (23, 46%), urine (16, 32%), endotracheal aspirate (6, 12%), sputum (3, 6%), intercostal drain fluid (1, 2%), and ascitic fluid (1, 2%). The median (interquartile range) age of the patients from whom the samples were obtained was 43 (13, 85) years, of which 29 were males, and 21 were females, with a male-to-female ratio of 1.4:1.

Phylogroups, multilocus sequence typing (MLST), and serotypes

Specific MLST types belong to a particular phylogroup (Table 1). The majority of the test isolates (25, 50%) belonged to phylogroup A, which has sequence types ST167 (15, 30%) and ST361 (4, 8%) as predominant sequence types as per the Achtman MLST typing scheme. Phylogroup A was followed by Phylogroup C (9, 18%), which comprised ST410 (4, 8%) and ST2851 (3, 6%) as predominant sequence type. The most common serotypes were O89:H9 (16, 32%), followed by O9:H30 (4, 8%), O102:H6 (3, 6%), and O100:H18 (3, 6%), respectively.

**Table 1:** Distribution of MLST sequence types and phylogroups MLST: multilocus sequence typing

Phylogroup	MLST types	n (%)
A	ST167	15 (30%)
	ST361	4 (8%)
	ST could not be ascertained	1 (2%)
	ST1702	1 (2%)
	ST2450	1 (2%)
	ST34	1 (2%)
	ST976	1 (2%)
C	ST410	4 (8%)
	ST2851	3 (6%)
	ST88	2 (4%)
F	ST648	5 (10%)
	ST6870	1 (2%)
B1	ST448	2 (4%)
	ST2083	1 (2%)
	ST2473	1 (2%)
	ST8346	1 (2%)
	ST940	1 (2%)
D	ST405	3 (6%)
	ST38	1 (2%)
B2	ST131	1 (2%)

Antibiotic test results

More than 45 (90%) isolates were MDR, all 50 (100%) showed resistance to penicillins, cephalosporins, and fluoroquinolones; more than 45 (90%) were resistant to β-lactam/β-lactamase inhibitor combinations and folate pathway inhibitors, and more than 35 (70%) were resistant to carbapenems. Resistance to aminoglycosides was substantial, with 33 (66%) isolates resistant to amikacin and 40 (80%) to gentamicin. However, tigecycline retained high activity, with 47 (94%) isolates being susceptible, while all 50 (100%) isolates demonstrated susceptibility to colistin, reported as intermediate in accordance with CLSI guidelines (Table 2).

**Table 2:** Percentage antibiotic susceptibility pattern of 50 Escherichia coli isolates by VITEK 2 Compact ID/AST system S: sensitive; I: intermediate; R: resistant; ID: identification; AST: antibiotic susceptibility testing; #: urinary antibiotics reported for 16 isolates

Antibiotics	Antibiotic susceptibility pattern by VITEK 2 Compact ID/AST system n (%)		
	S	I	R
Amikacin	17 (34%)	-	33 (66%)
Gentamicin	10 (20%)	-	40 (80%)
Piperacillin-tazobactam		2 (4%)	46 (92%)
Amoxicillin-clavulanate	2 (4%)	1 (2%)	47 (94%)
Cefoperazone-sulbactam	2 (4%)	4 (8%)	44 (88%)
Ciprofloxacin	-	-	50 (100%)
Ceftriaxone	-	-	50 (100%)
Cefepime	2 (4%)	-	48 (96%)
Ceftriaxone	-	-	50 (100%)
Cefuroxime	-		50 (100%)
Imipenem	13 (26%)	-	37 (74%)
Meropenem	14 (28%)	-	36 (72%)
Ertapenem	12 (24%)	2 (4%)	36 (72%)
Co-trimoxazole	2 (4%)	-	48 (96%)
Ampicillin	-	-	50 (100%)
Colistin	-	50 (100%)	-
Tigecycline	47 (94%)	-	3 (6%)
Urinary antibiotics (n = 16)			
Nitrofurantoin #	4/16 (25%)	7/16 (44%)	5/16 (31%)
Nalidixic acid #	-	-	16/16 (100%)

For 16 urinary isolates, complete resistance was noted to nalidixic acid in all 16 (100%) isolates, while nitrofurantoin showed variable activity, with four (25%) being sensitive, seven (44%) being intermediate, and five (31%) being resistant. The overall resistance pattern suggests the dominance of MDR *E. coli* strains with limited therapeutic options, especially in fluoroquinolones, third-generation cephalosporins, and carbapenems. The comparative analysis of phenotypic and genotypic AMR among 50 clinical *E. coli* isolates revealed a high prevalence of multidrug resistance (Table 3). Phenotypic resistance to fluoroquinolones, β-lactams, and folate pathway inhibitors was observed in 50 (100%), 50 (100%), and 48 (96%) isolates, respectively, which was in complete or near-complete concordance with genotypic findings. Genomic screening identified widespread mutations in quinolone resistance-determining regions (*gyrA, parC, parE*) and plasmid-mediated quinolone resistance genes (*qnrB1, qnrS1, qepA8*), corroborating universal fluoroquinolone resistance. Similarly, β-lactam resistance was mediated by an array of extended-spectrum β-lactamases (ESBLs) and carbapenemases, including *bla_CTX-M_, bla_TEM_, bla_OXA-1_*, and *bla_NDM_* variants. Carbapenem resistance was phenotypically observed in 37 (74%) isolates, aligning with the presence of *bla_NDM-1_, bla_NDM-5_,* and *bla_OXA-181_* in 38 (76%) strains. 

**Table 3:** A comparative summary of phenotypic and genotypic antimicrobial resistance profile of 50 Escherichia coli isolates

Antibiotic class	Antibiotic resistance genes detected by whole-genome sequencing	Phenotypic antimicrobial resistance n (%)	Genotypic antimicrobial resistance n (%)
Aminoglycosides (amikacin, gentamicin)	aac(6')-Ib3;aac(3)-IId;aac(3)-IIe;rmtC;rmtB;rmtB1;armA;aadA2;aadA5;aadA16;aadA1;aph(3'')-Ib;aph(6)-Id;aph(3')-Ia;armA;ant(2'')-Ia	40 (80%)	49 (98%)
Fluoroquinolones (ciprofloxacin)	parC_S80I;parC_A108V;parC_E84V;parE_S458A;parE_S458T;parE_E460D;parE_I529L;gyrA_D87N;gyrA_S83L;qepA8;qnrB4;qnrS1;qnrB1	50 (100%)	50 (100%)
Beta-lactams	bla_CMY;_bla_CMY__-145;_bla_CMY__-42;_bla_CMY-148;_bla_CMY-2;_bla_TEM;_bla_TEM-1_^;^bla_NDM;_bla_NDM-5;_bla_NDM-1;_bla_DHA;_bla_DHA-1;_bla_CTX-M-15;_bla_CTX-M-27;_bla_CTX-M-55;_bla_OXA-1;_bla_OXA-181;_bla_FONA_;ftsI_N337NYRIN;ftsI_I336IKYRI	50 (100%)	50 (100%)
Carbapenems	bla_OXA-181;_bla_NDM-5;_bla_NDM;_bla_NDM-1_	37 (74%)	38 (76%)
Folate pathway inhibitors (trimethoprim/sulfamethoxazole)	sul1;sul2;sul3;dfrA1;dfrA12;dfrA17;dfrA14;dfrA27;dfrA5	48 (96%)	48 (96%)
Polymyxins (colistin)	pmrB_Y358N;pmrB_E123D	0 (0%)	16 (32%)
Tetracycline*	tetA;tetB	3 (6%)	38 (76%)
Chloramphenicol*	catA1;catB3;catA3;cmlA6;cmlA5	24 (48%)	24 (48%)
Macrolides	mph(E);mph(A);erm(B);ere(A)	48 (96%)	49 (98%)

Phenotypically, 40 (80%) isolates were resistant to aminoglycosides, but 49 (98%) isolates carried aminoglycoside-modifying enzyme genes such as *aac(6')-Ib3, aadA1, rmtB, and armA*. In contrast, notable genotype-phenotype discordance was identified in polymyxin and tetracycline resistance. Despite the presence of *pmrB* mutations associated with colistin resistance in 16 (32%) isolates, none exhibited phenotypic resistance. Similarly, *tetA* and *tetB* genes were detected in 38 (76%) isolates, yet only three (6%) demonstrated phenotypic resistance. Resistance to chloramphenicol and macrolides demonstrated moderate-to-high correlation between genotype and phenotype, with *cat* and *cmlA* genes, and *mph(A) and* *erm(B)* genes, respectively, being frequently encountered. 

The analysis of AMR genes across sequence types revealed that the *bla_CTX-M_* group genes were the most widely distributed, particularly in ST131 (Phylogroup B2), the globally dominant ESBL clone. Carbapenemase genes such as *bla_NDM_* and* bla_OXA-48-like_* were concentrated in high-risk clones, including ST405/ Phylogroup D, ST410/ Phylogroup C, and ST167/Phylogroup A, often coexisting with ESBL determinants. The plasmid-mediated *qnr* genes were commonly found in ST131, ST405, and ST648, contributing to fluoroquinolone resistance, while the *mcr* gene, conferring colistin resistance, appeared sporadically in ST167 and ST410 (Table 4). 

**Table 4:** Predominant high-risk Escherichia coli clones with AMR, virulence and plasmids

ST (clone)	Phylogroup	Plasmid(s) involved	Major AMR genes	Major virulence genes	Clinical significance
ST167	A	IncF; ColRNAI-1	bla_NDM-1,5; _bla_CMY-42; _bla_TEM; _bla_CTX-M-15; _bla_OX__A__-1_	*fim, pap, afa *(adherence)*; iutA, fyuA, iroN *(iron uptake)	Dominant MDR clone in Asia; hybrid UPEC-like pathotype with high resistance
ST131	B2	IncFII; IncFIA	bla_CTX-M-27_; gyr-A	*pap, fim *(adherence)*; iuc, fep, ent *(iron uptake)	Pandemic clone; classic ExPEC/UPEC with high AMR
ST648	F	IncF; IncI1	bla_CMY-42; _bla_CTX-M-15; _bla_OX__A__-1;_bla_NDM-5_	*afa, yag *(adherence)*; fep *(iron uptake)*; ybt *(high pathogenicity island (HPI))	Emerging clone, globally spreading
ST405	D	IncF; Col (BS512)	bla_NDM-5; _bla_CTX-M-15; _bla_TEM-1_	*ybt *(HPI)*; yag, pap, fim *(adherence)*; iuc, fep, ent *(iron uptake)	Reported increasingly in India; linked to bloodstream infections

Virulence factors 

ST167 (Phylogroup A, 15 (30%))

ST167 (Phylogroup A, 15 (30%)) carried genes for adherence such as *fim* (type 1 fimbriae), *pap* (P fimbriae), and *afa *(afimbrial adhesin) along with iron uptake systems *iutA* (aerobactin receptor), *fyuA* (yersiniabactin receptor), and *iroN *(salmochelin siderophore receptor), suggestive of the uropathogenic *E. coli *(UPEC) pathotype.** **

ST648 (Phylogroup F, 5 (10%))

ST648 (Phylogroup F, 5 (10%)) contained multiple adherence and siderophore receptor genes (e.g., iroN, fyuA, iutA), again suggesting the potential of UPEC/ExPEC. 

ST410 (Phylogroup C, 4 (8%))

ST410 (Phylogroup C, 4 (8%)) is strongly represented by iron uptake genes such as fyuA (yersiniabactin receptor) and chuA (heme uptake system), along with invasion determinants, aligning with ExPEC/UPEC traits. 

ST405 (Phylogroup D, 3 (6%))

ST405 (Phylogroup D, 3 (6%)) exhibited fimbrial adherence genes (e.g., *fim, pap*) and iron acquisition systems, consistent with UPEC, and is also well-recognized as an extraintestinal pathogenic *E. coli *(ExPEC) lineage. 

No classical diarrheagenic *E. coli* pathotypes, such as enterotoxigenic *E. coli* (ETEC; heat-labile and heat-stable enterotoxins), Shiga toxin-producing *E. coli* (STEC; *stx1, stx2* genes), or enteroinvasive *E. coli* (EIEC; *ipaH* gene), were detected in the isolates. This indicates that the observed virulence profiles were primarily associated with ExPEC rather than classical diarrheal disease strains.

Plasmid profile

**Figure 1:**
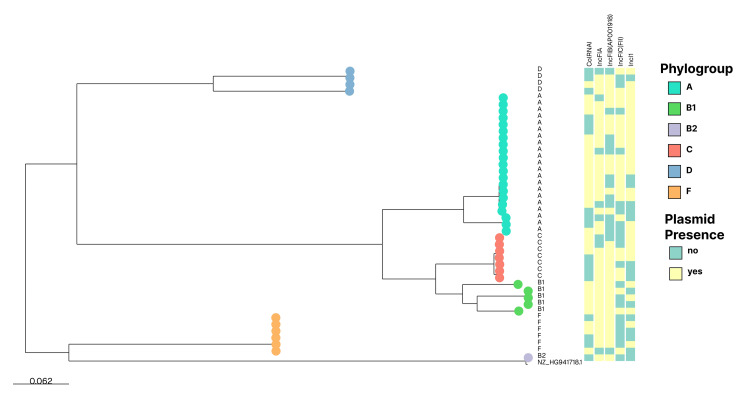
Plasmid profile of 50 Escherichia coli isolates as per phylogroups

Among 50 *E. coli* isolates, ST167 (15, 30%) showed the highest plasmid diversity, frequently carrying IncFIA_1 (13, 26%), IncFIB (AP001918)_1 (9, 18%), IncFIC(FII)_1 (14, 28%), and IncI1_1_Alpha (12, 24%). IncF-type replicons (FIA, FIB, FIC, FII) were the most prevalent, particularly IncFIC (FII)_1 (20, 40%) and IncFIA_1 (17, 34%). Multiple replicons were often detected in isolates from ST410/Phylogroup C, ST361/Phylogroup A, and ST648/Phylogroup F, indicating high gene transfer potential (Figure 1).

ColRNAI_1 was the next most prevalent replicon type, found in 20 (40%) isolates, suggesting its wide distribution across different STs, particularly ST167, ST2851, ST648, and others.

## Discussion

The present study undertakes a comprehensive phenotypic and genotypic characterization of 50 *E. coli *clinical isolates using WGS. The findings reveal significant AMR, notable phylogenetic diversity, and the widespread presence of virulence and plasmid-borne elements, thereby underscoring the public health implications of MDR *E. coli* in a tertiary care setting in North India.

Phylogroup diversity and sequence types

Phylogroup A has historically been linked to commensal strains with reduced virulence, but our data suggest that MDR clones from this group may have acquired significant resistance and virulence traits through gene and plasmid acquisition.

Sequence typing identified ST167 (15, 30%) as the most dominant lineage in our cohort. ST167 is a high-risk clone recognized for its role in the dissemination of carbapenemase genes such as *bla_NDM_* and *bla_OXA-48_*. In our study, carbapenemase genes such as* bla_NDM_* and *bla_CMY_* were found to be associated with ST167 [8,9]. Other notable sequence types identified include ST648 and ST410, both of which have been associated with MDR phenotypes and extraintestinal infections, i.e., from pus and urine samples. The presence of these clones in our dataset suggests clonal expansion and persistence within hospital environments, facilitated by strong selective pressure from empirical antibiotic use.

AMR: phenotypic and genotypic correlation

Phenotypic susceptibility testing revealed high levels of resistance across several antibiotic classes, with universal resistance to ciprofloxacin, ceftriaxone, and ampicillin. This is consistent with national AMR trends reported by the Indian Council of Medical Research (ICMR), which shows increasing resistance to fluoroquinolones and β-lactams among *E. coli *isolates in tertiary care centers [3]. Resistance to carbapenems in our data set is of grave concern, as these are considered last-resort agents. Similar high-level resistance has been reported in ICU settings in India [9]. The coexistence of multiple ESBL and carbapenemase genes in individual isolates highlights the expanding resistome of *E. coli *in India [7,8].

Notably, phenotypic resistance to fluoroquinolones and β-lactams was supported by the presence of these mutations through genomic analysis. But for tetracyclines and polymyxins, while more than 70% of isolates harbored genes *tetA* and *tetB*, only a few showed phenotypic resistance; this could reflect nonfunctional mutations, downregulated gene expression, or compensatory regulatory mechanisms as previously suggested in similar studies [20-23]. Such discrepancies underscore the importance of integrating both phenotypic and genotypic methods for accurate resistance profiling. From a therapeutic standpoint, the susceptibility to colistin and tigecycline, retained in all 50 (100%) and 47 (94%) isolates, respectively, offers some reassurance. However, reliance on these last-resort drugs may accelerate the emergence of pan-resistant strains. 

Virulence factors and pathogenic potential

Virulence gene profiling revealed substantial heterogeneity across the isolates, reflecting the genomic plasticity of *E. coli*. The presence of classical adhesins, *fimH, fimA*, and *ecp *operon genes across multiple STs confirms their role in early colonization and biofilm formation [23]. Notably, high-risk clones like ST131 and ST405 in pus samples carried a broad array of adhesin and fimbrial adhesin genes, such as afaA and afaC, indicating enhanced epithelial adherence and colonization potential [24].

The co-presence of *aslA, kpsD*, and *kpsM *in isolates belonging to ST131 and ST405 may enhance their ability to breach host barriers, invade the bloodstream, and cause sepsis or meningitis [8]. The observed clustering of invasion genes in these STs correlates with previous reports that associate these genes with severe clinical outcomes [25].

Iron acquisition systems were ubiquitous, with the enterobactin pathway genes (*ent, fep, fes*) present in the majority of the isolates. This siderophore system is considered the primary iron uptake mechanism in *E. coli* and contributes to its survival in iron-limited environments such as the urinary tract and bloodstream. The presence of yersiniabactin operons (*ybtA-X*) in more than 60% of the isolates, particularly among ST167, ST648, and ST405, is significant. These genes are typically located on integrative conjugative elements (ICEs) and contribute to pathogenicity, particularly in urosepsis and neonatal meningitis [26,27]. Effector delivery system genes, particularly the *esp* gene family, were identified in 90% of isolates. These genes are typically associated with the type III secretion system (T3SS) and play a role in host immune evasion and cytotoxicity [28]. The consistent presence of *espX1, espX4*, and *espY1* in phylogroups A and F suggests a possible functional T3SS repertoire, enhancing the virulence potential of these clones.

Toxin genes were relatively infrequent, with only 11 (22%) isolates carrying at least one such gene; *sat *(secreted autotransporter toxin) was the most common, followed by *astA* (EAST-1). This limited prevalence of classical toxins supports the notion that extraintestinal virulence in *E. coli* is often mediated more by adhesins, invasins, and iron acquisition systems than by cytotoxins alone [5].

Plasmid content and horizontal gene transfer

Plasmid analysis revealed significant diversity, with IncF-type plasmids being the most prevalent. These plasmids are well-known for co-harboring both resistance and virulence genes and play a pivotal role in the persistence and dissemination of high-risk clones. IncF plasmids are major reservoirs of both AMR and virulence genes in high-risk clones (ST167, ST648, ST405, ST131) as observed by other authors [29]. The widespread presence of such plasmids suggests that, beyond encoding resistance, they may play a role in niche adaptation and chronic infection establishment.

Clinical and public health implications

The convergence of resistance and virulence traits in the same isolates, particularly in ST167, ST648, and ST131, represents a serious clinical threat. Such convergence facilitates the emergence of strains that are not only difficult to treat but also capable of causing severe infections. This phenomenon has been increasingly reported across Asia and Europe, where clone evolution is constantly occurring due to antibiotic misuse, inadequate surveillance, and high infection burden [14].

The findings of this study are aligned with previous national efforts that call for the inclusion of WGS in routine microbiological surveillance [30]. WGS provides high-resolution data on pathogen lineage, resistance mechanisms, and outbreak tracking. 

Limitations and future directions

Although this study provides valuable insights, several limitations must be acknowledged. The relatively small sample size and single-center design may limit the generalizability of epidemiological inferences; however, the findings establish a strong baseline for future multicentric investigations. Functional assays to validate gene expression and confirm resistance mechanisms were not undertaken, which could have further clarified genotype-phenotype relationships. Clinical outcomes were not assessed, and thus, the correlation between patient outcomes and specific MLST types carrying virulence or AMR determinants could not be established, as the dataset represented a one-time isolate collection without longitudinal follow-up. Additionally, infection control interventions were not formulated based on the genomic surveillance data, although the findings highlight the potential of such data to inform and strengthen infection prevention strategies in the future.

Future research should incorporate longitudinal surveillance of circulating *E. coli* clones, integration of transcriptomic analyses, and exploration of host immune responses that shape pathogenicity. Expanded plasmid profile studies may also help identify novel resistance cassettes and mobile genetic elements contributing to the MDR burden.

## Conclusions

The concurrent presence of resistance and virulence genes, particularly within high-risk clones such as ST167, ST131, and ST648, represents a significant concern, as it predisposes to infections that are both difficult to treat and potentially associated with poorer clinical outcomes. From an infection control perspective, timely identification of such high-risk clones carrying transferable resistance determinants enables healthcare facilities to implement focused interventions, including enhanced contact precautions, targeted decolonization strategies, and adjustments in empirical treatment protocols.
